# Contrasting photosynthesis and photoinhibition in tetraploid and its autodiploid honeysuckle (*Lonicera japonica* Thunb.) under salt stress

**DOI:** 10.3389/fpls.2015.00227

**Published:** 2015-04-09

**Authors:** Kun Yan, Congwen Wu, Lihua Zhang, Xiaobing Chen

**Affiliations:** ^1^Key Laboratory of Coastal Environmental Processes and Ecological Remediation, Yantai Institute of Coastal Zone Research, Chinese Academy of SciencesYantai, China; ^2^Shandong Provincial Key Laboratory of Coastal Environmental Processes, Yantai Institute of Coastal Zone Research, Chinese Academy of SciencesYantai, China; ^3^Academy of Life Sciences, Yantai UniversityYantai, China

**Keywords:** honeysuckle, Na^+^, photosystem, photosynthetic electron transport, polyploidy

## Abstract

Honeysuckle (*Lonicera japonica* Thunb.) is a popular landscape plant. This study was to explore leaf photosynthetic characterization with emphasis on the coordination between photosystem II (PSII) and photosystem I (PSI) in tetraploid and its autodiploid honeysuckle (TH and DH) upon salt stress (300 mM NaCl). Leaf photosynthetic rate and carboxylation efficiency in DH and TH were significantly decreased under salt stress, and the decrease was greater in DH. PSII photoinhibition was induced in DH under salt stress, as the maximum quantum yield of PSII (Fv/Fm) was significantly decreased. PSII photoinhibition declined electron flow to PSI, but did not prevent PSI photoinhibition, as the maximal photochemical capacity of PSI (MR/MR_0_) was significantly decreased by salt stress. According to the significant decrease in PSI oxidation amplitude in the first 1 s red illumination, PSI photoinhibition was more severe than PSII photoinhibition. As a result, PSII and PSI coordination was destroyed. Comparatively, salt-induced photoinhibition did not occur in TH, as no significant change was observed in Fv/Fm and MR/MR_0_. Consequently, PSII and PSI coordination was not significantly affected by salt stress. In conclusion, TH maintained normal coordination between PSII and PSI by preventing photoinhibition and exhibited higher leaf photosynthetic activity than DH under salt stress. Compared with DH, lower leaf ionic toxicity due to greater root Na^+^ extrusion and restriction of Na^+^ transport to leaf might be responsible for maintaining higher leaf photosynthetic capacity in TH under salt stress.

## Introduction

Salinity is one of the main abiotic stresses which reduce plant growth and development. Salt stress can damage biological macromolecules and interfere with metabolisms in plant cells by inducing osmotic stress and ionic toxicity (Munns and Tester, 2008). Photosynthesis closely correlates with plant growth and is sensitive to salt stress, and photosynthetic capacity is an important criterion for diagnosing plant adaptability to salinity (Kalaji and Pietkiewicz, 1993; Kalaji et al., 2011).

Up to now, the effects of salt stress on plant photosynthesis have been extensively studied. The decreased CO_2_ availability due to the diffusion limitation of stomata is considered as the initial negative effect of salt stress on photosynthesis (Loreto et al., 2003; Chaves et al., 2009). Rubisco is a crucial enzyme in CO_2_ fixation process. Salt stress can suppress Rubisco activity by reducing Rubisco content and activation, and lead to the decline of CO_2_ fixation (Feng et al., 2007; Lu et al., 2009). The inhibition on CO_2_ assimilation will increase accumulation of reducing equivalents in the form of NADPH, underlie over-reduction of photosynthetic electron transport chain and elevate excitation pressure in chloroplast. If the excess excitation energy cannot be dissipated, ROS production will be increased and then bring about photoinhibition (Muller et al., 2001; Takahashi and Murata, 2008). NPQ plays a major role in photoprotection, as it can dissipate the excess excitation energy from light as heat and lower the creation of ROS (Bilger and Bjorkman, 1990; Carbonera et al., 2012; Brestic et al., 2014). PSII photoinhibition is a result of the imbalance between PSII photodamage and the repair of such damage (Murata et al., 2007). PSII photodamage is initiated by the direct effect of light on the OEC, and ROS inhibit the repair of photodamaged PSII mainly by suppressing the synthesis of D1 protein. In the existing studies, it is not consistent whether salt stress can induce PSII photoinhibition, possibly due to the different plant materials and salt treatment protocols (Chen et al., 2004; Netondo et al., 2004; Kalaji et al., 2011; Hussain et al., 2012; Chen et al., 2013). In contrast, few studies pay attention to the salt effects on PSI capacity in plants (Stepien and Johnson, 2009). PSI photoinhibition is induced by ROS produced at the acceptor side of PSI through Mehler reaction *in vivo* (Sonoike, 2011). The electron flow from PSII is essential for PSI photoinhibition, and the addition of 3-(3,4-dichlorophenyl)-1,1-dimethylurea, an inhibitor of PSII primary electron acceptor oxidation, can suppress PSI photoinhibition and help PSI recovery after photoinhibition (Sonoike, 1996; Zhang et al., 2011). PSI photoinhibition is more dangerous than PSII photoinhibition because of the difficult recovery process of PSI (Kudoh and Sonoike, 2002; Zhang and Scheller, 2004). Therefore, rapid PSII photoinhibition under high temperature or high light stress protects PSI from photoinhibition by restricting the electron flow to PSI (Herrmann et al., 1997; Yan et al., 2013a,b; Zivcak et al., 2014). PSI photoinhibition usually arises under chilling stress with low light because of the limited restriction on electron flow to PSI, and in particular, rapid recovery of PSII after chilling stress is detrimental to the recovery of PSI (Zhang et al., 2011). Thus, PSII and PSI coordination plays an important role in protecting PSI or even the whole photosynthetic apparatus. However, it is still largely unknown about the interaction between PSII and PSI in plants under salt stress.

Polyploidy indicates the doubling of chromosomes of a single species or the hybrids between two species, and polyploidy usually can enhance plant tolerance to abiotic stresses. In contrast to the diploid, polyploid black locust, turnip and *Arabidopsis* exhibited stronger salt tolerance with less decrease in biomass and lower accumulation of leaf Na^+^ under salt stress (Meng et al., 2011; Chao et al., 2013; Wang et al., 2013). On the contrary, Mouhaya et al. (2010) reported that tetraploid citrus accumulated more Na^+^ in the leaf and showed greater decrease in PSII capacity than diploid citrus. Therefore, it is not confirmed whether polyploidy can enhance plant salt tolerance. Photosynthetic characterization, especially for PSII and PSI interaction, has not been deeply explored in polyploid plant under salt stress.

Honeysuckle, a twining semi-evergreen vine, is a popular landscape plant with high environmental adaptability and distributed widely in temperate and tropical regions. TH has less whole plant leaf area, higher leaf mass per unit area, thicker epidermis, and palisade tissue as well as denser pubescence compared with its diploid progenitor, and the stronger drought tolerance in TH originates from these morphological characterizations (Li et al., 2009). In this study, we intended to reveal photosynthetic characterization with emphasis on PSII and PSI coordination in tetraploid and its autodiploid honeysuckle and discriminate their salt tolerance. Our study can deepen the knowledge of salt tolerance mechanism in polyploid plants and may provide a reference for cultivar selection in saline land greening.

## Materials and Methods

### Plant Material and Treatment

Stem cuttings from two cultivars of honeysuckle, tetraploid (Jiufengyihao, 36 chromosomes) and its diploid progenitor (Damaohua, 18 chromosomes) were bought from jiujianpeng agricultural technology limited company (Pingyi, Shandong, China). The tetraploid cultivar was bred by treating the stem tips of a diploid cultivar with colchicines (Li et al., 2009). The cuttings were planted in a nursery in November, 2013, and then transplanted to the plastic pots filled with quartz sand in April, 2014. The plants were watered with Hoagland solution (pH 5.7) and placed in climatic chambers (Huier, China). The photon flux density was approximately 200 μmol m^-2^ s^-1^ (12 h per day from 07:00 to 19:00) in the chambers. Day/night temperature and humidity were controlled at 25/18°C and 65% in the chambers. After 60 days, healthy and uniform plants were selected for salt treatments. NaCl was added to nutrient solution incrementally by 50 mM step every day to provide final concentration of 300 mM. Nutrient solution without adding NaCl was used to cultivate the control plants. The expanded leaves from the middle of a shoot were sampled for measuring photosynthetic parameters and MDA content. After salt stress for 15 days, roots, and leaves were harvested, rinsed with deionized water and wiped with tissues. Thereby, they were dried at 105°C for 10 min, and then dried at 70°C to constant weight for measuring Na^+^ content.

### Measurements of Gas Exchange and Chlorophyll Fluorescence Parameters

Gas exchange and modulated chlorophyll fluorescence parameters were simultaneously detected by using an open photosynthetic system (LI-6400XTR, Li-Cor, Lincoln, NE, USA) equipped with a fluorescence leaf chamber (6400-40 LCF, Li-Cor).

The leaves were dark-adapted for 30 min before the measurements. The minimal fluorescence level in the dark adapted state was measured using a modulated pulse (<0.05 μmol m^-2^ s^-1^ for 1.8 s). Maximal fluorescence was measured after applying a saturating actinic light pulse of 8000 μmol m^-2^ s^-1^ for 0.7 s. Subsequently, actinic light intensity was altered to 1000 μmol m^-2^ s^-1^ in leaf cuvette and then maintained for about 30 min. The temperature, CO_2_ concentration and relative humidity were, respectively, set at 25°C, 400 μmol mol^-1^ and 65% in the leaf cuvette. Pn, g_s_ and Ci were simultaneously recorded. In addition, steady-state fluorescence yield was also recorded. Then, a saturating actinic light pulse of 8000 μmol m^-2^ s^-1^ for 0.7 s was used to produce maximum fluorescence yield by temporarily inhibiting PSII photochemistry, and the minimum fluorescence in the steady state was determined during a brief interruption of actinic light irradiation in the presence of far-red light (λ = 740 nm). At last, ΦPSII and NPQ were calculated (Maxwell and Johnson, 2000).

For the measurement of CE, photon flux density and temperature were set at 1000 μmol m^-2^ s^-1^ and 25°C in the leaf cuvette. Pn was measured under CO_2_ concentrations in a sequence of 700, 500, 400, 300, 200, 150, 100, and 50 μmol mol^-1^. The leaves were kept under each level of CO_2_ concentration for 4 min to let leaves reach steady-state photosynthesis, and Pn and Ci were then recorded. The correlation curve of Pn related to Ci was established, and CE was calculated from the linear portion of Pn-Ci curve.

### Measurement of Chlorophyll *a* Fluorescence and Modulated 820 nm Reflection Transients

The measurements were made by using a multifunctional plant efficiency analyzer (MPEA, Hansatech, UK), and the operating mechanism of this instrument has been elucidated in detail (Strasser et al., 2010; Kalaji et al., 2012). The leaves were adapted in dark for 30 min before the measurement (Kalaji et al., 2014b). Thereafter, the leaves were orderly illuminated with 1 s red light (627 nm, 5000 μmol photons m^-2^ s^-1^), 10 s far red light (735 nm, 200 μmol photons m^-2^ s^-1^) and 2 s red light (627 nm, 5000 μmol photons m^-2^ s^-1^). Chlorophyll *a* fluorescence and modulated 820 nm reflection were simultaneously recorded during the illumination. Monitoring modulated reflection change near 820 nm is a very convenient way to follow the redox state of PSI. The relative value of the maximal difference of 820 nm reflection during the last 2 s red illumination was used to indicate ΔMR/MR_0_ (Schansker et al., 2003). MR_0_ is the value of 820 nm reflection at 0.7 ms (the first reliable MR measurement). ΔMR is the value of the maximal difference of 820 nm reflection at the last 2 s red light illumination.

Chlorophyll *a* fluorescence transients were quantified according to JIP test by using the following original data: (1) fluorescence intensity at 20 μs (Fo, when all reaction centers of PSII are open); (2) the maximum fluorescence intensity (Fm, when all reaction centers of PSII are closed) and (3) fluorescence intensities at 300 μs (F_k_, K step) and 2 ms (F_J_, J step). Using these original data, some parameters can be calculated for quantifying PSII behavior (Strasser et al., 2010). Fv/Fm, W_k_ and ETo/ABS was, respectively, calculated as: Fv/Fm = (Fm - Fo)/Fm, W_k_ = (F_k_ - Fo)/(F_J_ - Fo) and ETo/ABS = (Fm - F_J_)/Fm.

### Measurements of Na^+^ Content and Na^+^ Translocation Factor

The extraction of Na^+^ was performed according to Song et al. (2011). Deionized H_2_O (25 ml) was added to 0.1 g dried plant powder and boiled for 2 h. The supernatant was diluted 50 times with deionized H_2_O for measuring Na^+^ content by using an atomic absorption spectrophotometer (TAS-990, China). Na^+^ translocation factor was calculated as the ratio of Na^+^ concentration between leaves and roots.

### Measurement of Root Na^+^ Flux

Net Na^+^ flux was measured using NMT (NMT system BIO-IM, Younger, USA). The concentration gradients of the target ions were measured by moving the ion-selective microelectrode between two positions close to the plant material in a pre-set excursion (20 μm for excised roots in the present experiment). The ion fluxes were calculated based on the Fick’s law of diffusion.

Prepulled and silanized glass micropipettes (Xuyue Sci. and Tech., China) were firstly filled with a backfilling solution (100 mM NaCl) to a length of approximately 1 cm from the tip. Then the micropipettes were front filled with selective liquid ion-exchange cocktails (LIXs: Na, Sigma 71178). An Ag/AgCl wire electrode holder (Xuyue Sci. and Tech., China) was then inserted in the back of the electrode to make electrical contact with the electrolyte solution. DRIREF-2 (World Precision Instruments) was used as the reference electrode. Ion-selective electrodes were firstly calibrated before flux measurement using the following solutions: 5, 2, 0.5 mM Na^+^. Only electrodes with Nernstian slopes >50 mV/decade were used. Ion flux was calculated by Fick’s law of diffusion: J = –D(dc/dx) where J represents the ion flux in the x direction, dc/dx is the ion concentration gradient, and D is the ion diffusion constant in a particular medium. Data and image acquisition, preliminary processing, control of the three-dimensional electrode positioner, and stepper-motor-controlled fine focus of the microscope stage were performed with IM-Flux software.

**FIGURE 1:**
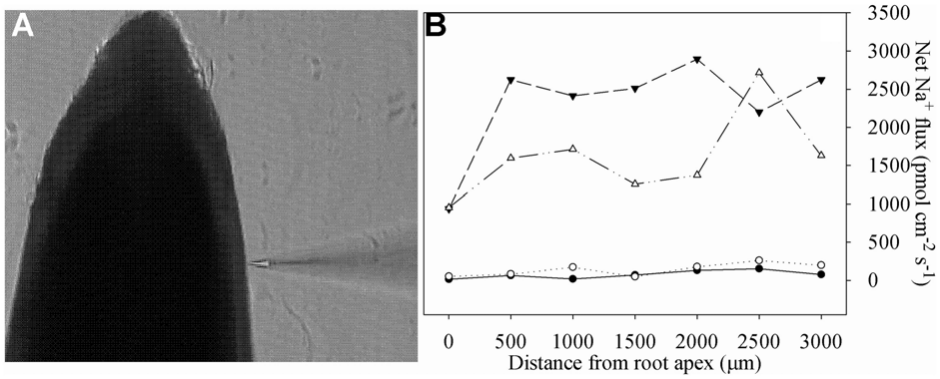
**The non-invasive ion-selective electrode closed to the root **(A)** and net Na^+^ fluxes in roots of tetraploid (closed symbols) and diploid (open symbols) honeysuckle exposed to 0 (circles) and 300 mM (triangles) NaCl (B).** Na^+^ fluxes were measured along root axes (0–3000 μm from the apex) at interval of 500 μm. Each point is the mean of five individual plants.

Newly developed root segments (5 cm from apex) were sampled, rinsed with deionized water and immediately incubated in the measuring solution to equilibrate for 30 min. Thereafter, roots were transferred to the measuring chamber containing 15 ml fresh measuring solution. Na^+^ measuring solutions were as follows: 0.1 mM KCl, 0.1 mM CaCl_2_, 0.1 mM MgCl_2_, 2 mM NaCl, 0.3 mM MES, pH 6.0 (adjusted with choline and HCl). After the roots were immobilized on the bottom, ion flux measurements were started. Ion flux measurements were started from the apex and went along the root axis until 3000 μm at interval of 500 μm. The measured root positions could be visualized and defined under the NMT microscope (**Figure 1A**). As shown in **Figure 1B**, Na^+^ eﬄux along the root axes was increased by salt stress, and according to these data, the average value of Na^+^ eﬄux was calculated and shown in **Table 1**.

**Table 1:** Leaf and root dry weight per plant, leaf and root Na^+^ content, Na^+^ translocation factor and mean of root Na^+^ flux in TH and DH exposed to 0 and 300 mM NaCl.

Parameters	TH (0 mM)	TH (300 mM)	DH (0 mM)	DH (300 mM)
Leaf DW(g/plant)	3.30 ± 0.24a	2.50 ± 0.11b	2.64 ± 0.17b	1.21 ± 0.21c
Root DW(g/plant)	0.98 ± 0.08a	0.65 ± 0.04b	0.86 ± 0.09ab	0.40 ± 0.04c
Leaf Na^+^ content (mg g^-1^ DW)	8.69 ± 0.88c	16.24 ± 1.06b	8.79 ± 0.76c	33.01 ± 1.76a
Leaf MDA content (mg g^-1^ DW)	0.68 ± 0.06b	0.69 ± 0.05b	0.70 ± 0.03b	1.17 ± 0.07a
Root Na^+^ content (mg g^-1^ DW)	14.75 ± 3.02c	26.15 ± 3.04b	13.16 ± 0.93c	34.78 ± 2.68a
Na^+^ translocation factor	1.91 ± 0.25b	2.28 ± 0.19b	2.03 ± 0.32b	2.93 ± 0.25a
Mean of root Na^+^ eﬄux (pmol cm^-2^ s^-1^)	75.36 ± 10.37c	2315.27 ± 242.62a	185.32 ± 56.23c	1605.03 ± 289.27b

DW indicates dry weight. Data in the table indicate the mean of five replicates (±SD). Within each row, means followed by the same letters are not significantly different at *P* < 0.05.

### Measurement of MDA Content

Leaf tissues (0.5 g) were ground under liquid nitrogen and then homogenized in 5 ml of 50 mM potassium phosphate buffer (pH 7.8). After centrifugation at 4°C and 13000 ×*g* for 10 min, the supernatant was prepared for the assay of MDA content (Yan et al., 2010).

### Statistical Analysis

One-way ANOVA was carried out by using SPSS 16.0 (SPSS Inc., Chicago, IL, USA) for all sets of data. The values presented are the means of measurements with five replicate plants, and comparisons of means were determined through LSD test. Difference was considered significant at *P* < 0.05.

## Results

### Effects of Salt Stress on Biomass, Na^+^ and MDA Content and Root Na^+^ Flux

Leaf and root dry weight per plant were significantly decreased, respectively, by 24.24 and 33.67% in TH and by 54.17 and 53.49% in DH after salt stress, and the decrease was greater in DH (**Table 1**). Salt stress significantly increased leaf and root Na^+^ content by 86.88 and 77.29% in TH, and the greater increase with 275.54 and 164.29% was found in DH. Root Na^+^ eﬄux was elevated by 10.27 fold in DH after salt stress, and the greater increase with 29.72 fold was found in TH. Na^+^ translocation factor was significantly increased by 44.33% in DH after salt stress, but no significant change was noted in TH, suggesting that Na^+^ transport to the leaf was restricted in TH. The extent of lipid peroxidation represented by MDA content reflects the state and integrity of membranes in plant cells (Blokhina et al., 2003; Yazici et al., 2007). Leaf MDA content was not significantly affected in TH after salt stress, whereas the significant increase was observed in DH.

### Effects of Salt Stress on Gas Exchange and Chlorophyll Fluorescence Parameters

**FIGURE 2:**
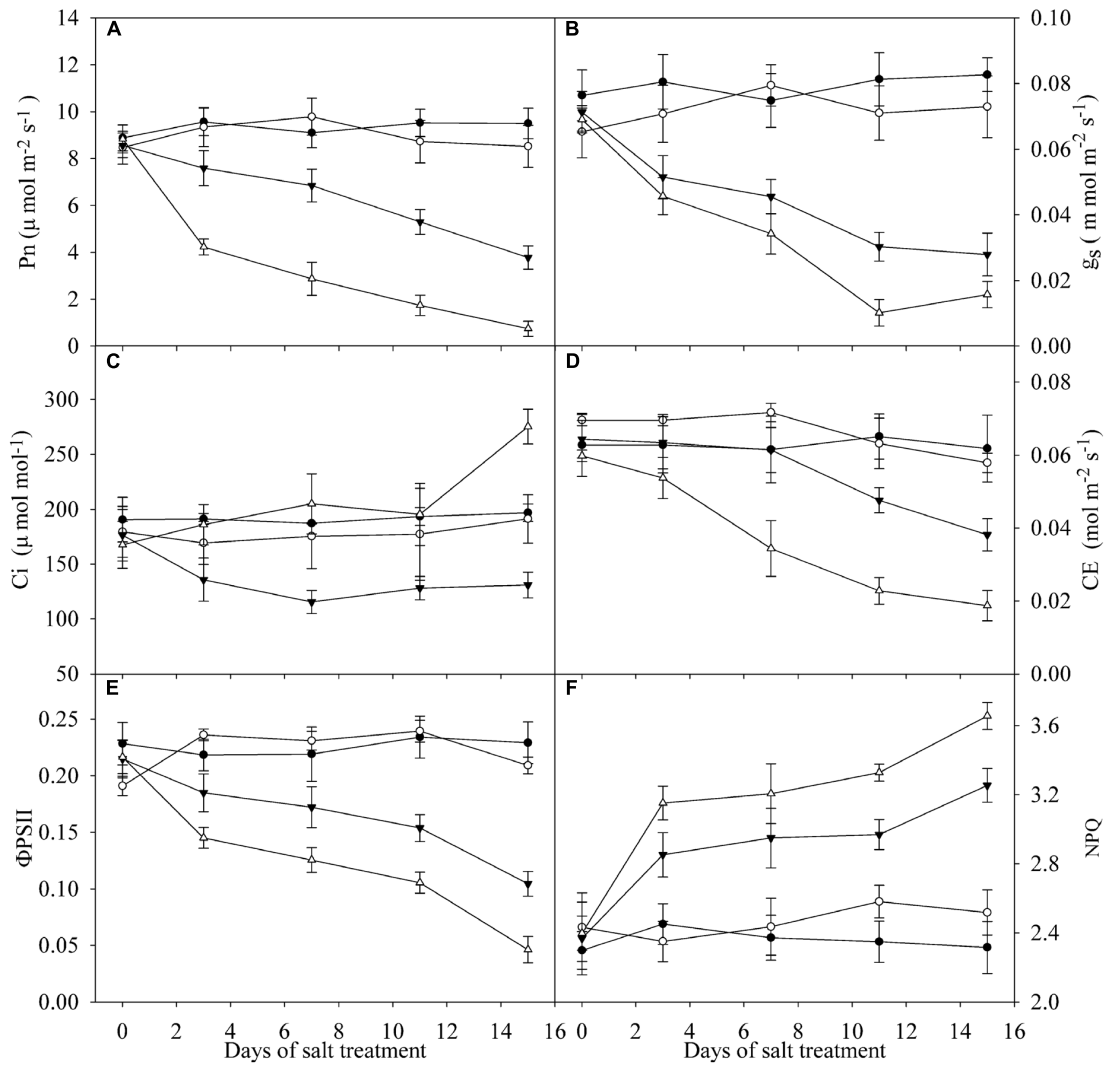
**Pn **(A)**, g_**s**_**(B)**, Ci **(C)**, CE **(D**), ΦPSII **(E)** and NPQ **(F)** in the leaves of tetraploid (closed symbols) and diploid (open symbols) honeysuckle exposed to 0 (circles) and 300 mM (triangles) NaCl.** Data in the figure indicate the mean of five replicates (±SD).

Pn, g_s_ and ΦPSII were significantly decreased in the leaves of TH and DH under salt stress, and the decrease was greater in DH than TH (**Figures 2A,B,E**). Under salt stress, Ci was lowered in the leaves of TH, but Ci in the leaves of DH was not significantly changed and even remarkably elevated at day 15 (**Figure 2C**). Under salt stress, CE in the leaves of DH was significantly decreased at day 3, whereas the significant decrease in CE was not recorded in the leaves of TH until day 11, and the decrease was lower than that in DH (**Figure 2D**). NPQ in the leaves of TH and DH was significantly increased by salt stress (**Figure 2F**).

### Effects of Salt Stress on Chlorophyll *a* Fluorescence and Modulated 820 nm Reflection Transients

**FIGURE 3:**
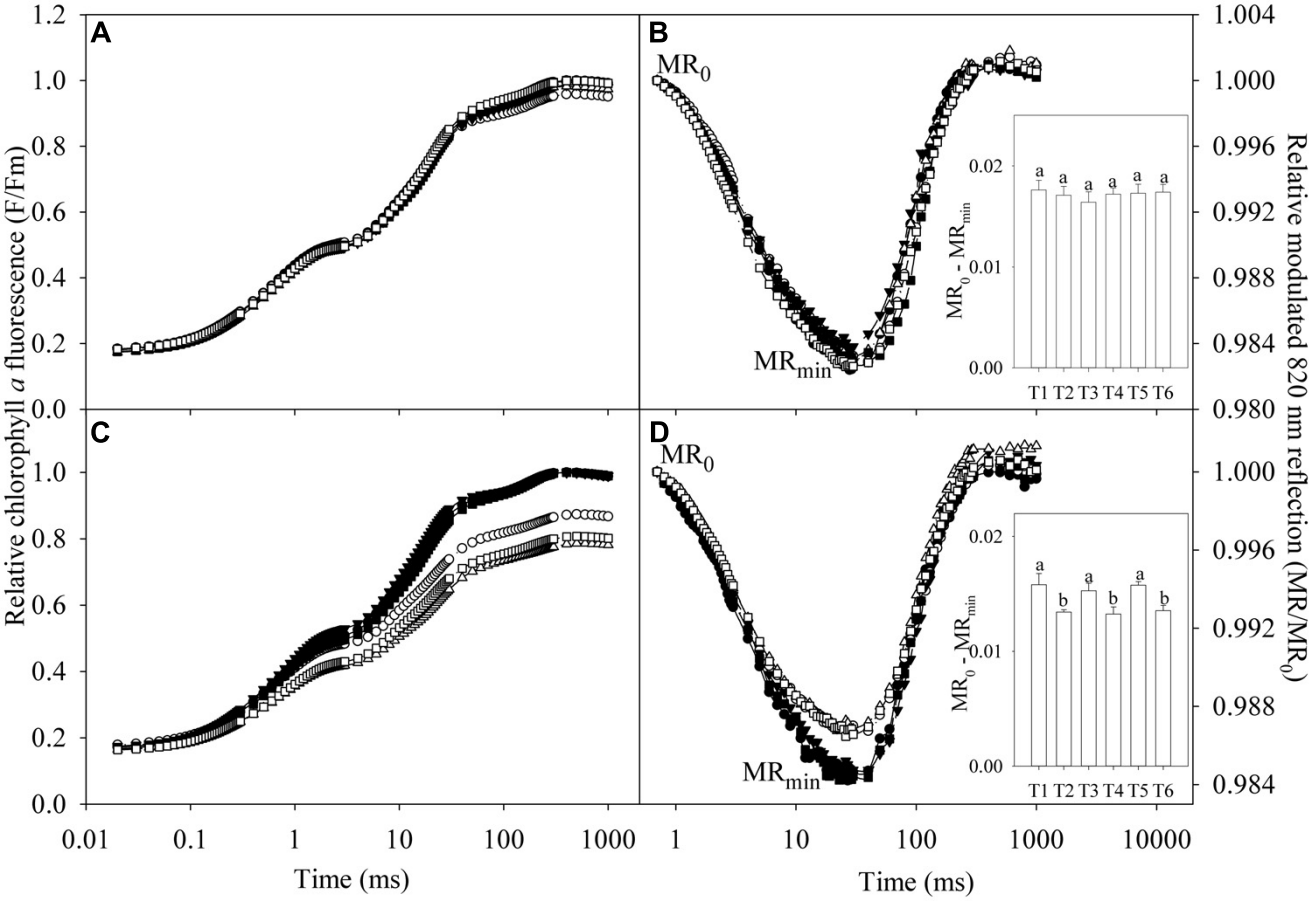
**Relative chlorophyll *a* fluorescence (F/Fm) and relative modulated 820 nm reflection (MR/MR_**0**_) during the first 1 s red illumination in the leaves of tetraploid **(A,B)** and diploid **(C,D)** honeysuckle exposed to 0 (closed symbols) and 300 mM (open symbols) NaCl at day 7 (circles), 11 (triangles), and 15 (squares).** F is chlorophyll *a* fluorescence intensity during the 1 s of red illumination and Fm is the maximal fluorescence intensity. MR_0_ is the value at the onset of actinic illumination (at 0.7 ms) and MR is the reflection signal during the 1 s of red illumination. MR_min_ is the minimal value of MR/MR_0_ at about 29 ms. T1, T2, T3, T4, T5, and T6, respectively, indicate the treatments with 0 and 300 mM NaCl for 7, 11, and 15 days. Data in the figure indicate the mean of five replicates. Different letters on error bars indicate significant difference at *P* < 0.05.

Salt stress did not obviously affect chlorophyll *a* fluorescence transients and 820 nm reflection transients in the leaves of TH (**Figures 3A,B**). Chlorophyll *a* fluorescence was declined under salt stress in the leaves of DH, indicating PSII capacity was negatively affected (**Figure 3C**). The 820 nm reflection signals are presented by MR/MR_0_ ratio, where MR_0_ is the value at the onset of actinic illumination (at 0.7 ms). Decrease in MR/MR_0_ from MR_0_ to the minimal value (MR_min_, at about 29 ms) reflects PSI oxidation process, and the oxidation amplitude in the first 1 s red illumination was expressed as MR_0_ - MR_min_. The minimal value of MR is a transitory steady state with equal oxidation and re-reduction rate of PSI. Subsequently, increase in MR/MR_0_ indicates PSI re-reduction driven by the electron flow from PSII. Thus, 820 nm reflection transient in the first 1 s red illumination was influenced by both PSII and PSI capacity and could reflect their coordination. The 820 nm reflection transient was obviously changed in the leaves of DH under salt stress, indicating the negative effects on PSII and PSI coordination. PSI oxidation amplitude was significantly decreased in the leaves of DH under salt stress, whereas no significant change occurred in the leaves of TH (The inserted panels of **Figures 3B,D**).

### Effects of Salt Stress on Fv/Fm, W_**k**_, ETo/ABS, and ΔMR/MR_0_

**FIGURE 4:**
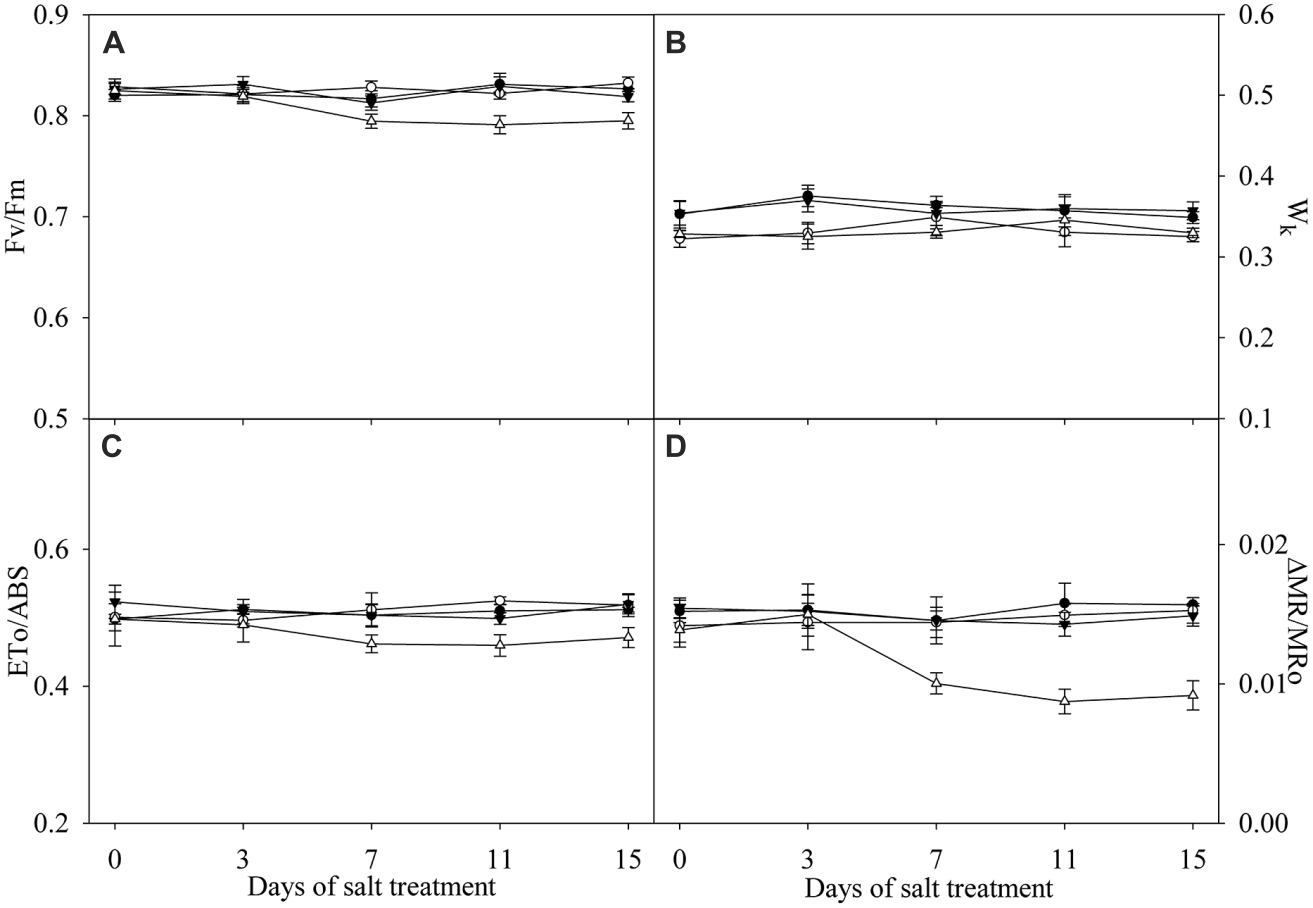
**Fv/Fm (A), Wk (B), ETo/ABS (C) and ΔMR/MR0 (D) in the leaves of tetraploid (closed symbols) and diploid (open symbols) honeysuckle exposed to 0 (circles) and 300 mM (triangles) NaCl.** Data in the figure indicate the mean of five replicates (±SD).

Under salt stress, Fv/Fm, W_k_, ETo/ABS, and MR/MR_0_ were not significantly affected in the leaves of TH (**Figure 4**). Significant decrease in Fv/Fm, ETo/ABS, and MR/MR_0_ was found in the leaves of DH after 7 days of salt stress (**Figures 4A,C,D**), indicating the occurrence of PSII and PSI photoinhibition. Increase in W_k_ with an elevated K step around 300 μs in chlorophyll *a* fluorescence transient is a specific indicator of injury on OEC (Strasser, 1997; Kalaji et al., 2014a). Insignificant change in W_k_ and no obvious K step indicated that OEC was not damaged in the leaves of DH and TH under salt stress (**Figures 3A,C** and 4B).

## Discussion

Plant photosynthesis and growth are commonly constrained in saline environment, and salt-tolerant plants can better adapt to salinity with less decrease in biomass and photosynthetic capacity (Kalaji and Nalborczyk, 1991; Stepien and Johnson, 2009; Chao et al., 2013; Aparicio et al., 2014). In this study, stronger salt tolerance was defined in TH, because TH maintained higher photosynthetic capacity and accumulated greater biomass than DH under salt stress (**Table 1**; **Figure 2**).

Photosynthetic rate, g_s_ and Ci in TH concomitantly declined under salt stress, suggesting the stomatal limitation on photosynthesis (Figures 2A–C; Farquhar and Sharkey, 1982). The declined g_s_ can serve as a protective way against salt-induced osmotic stress by reducing water loss from transpiration, but increases the stomatal limitation of photosynthesis (Chaves et al., 2009). TH has stronger osmotic adjusting ability than DH due to the morphological and anatomical characterizations (Li et al., 2009), and therefore, TH maintained higher leaf g_s_ under salt stress, which helped to alleviate stomatal limitation on photosynthesis. The salt-induced change in Ci was not coincident with Pn and g_s_ in DH (Figures 2A–C), suggesting that stomatal limitation did not play a major role in the decrease of Pn. CE positively correlates with Rubisco activity (Voncaemmerer and Farquhar, 1981). The declined CE indicated CO_2_ fixation was depressed, and the greater inhibition on CO_2_ fixation in DH possibly resulted in more severe oxidative stress on photosystem (**Figure 2D**). Allakhverdiev et al. (2002) reported that salt stress induced PSII photoinhibition in *Synechocystis* by inhibiting the repair of photodamaged PSII not by directly accelerating photodamage on OEC. In this study, PSII photoinhibition without injury on OEC in DH also implied the negative effects of salt stress on PSII repair (**Figures 4A,B**). As the traditional viewpoint, PSII is more susceptible to abiotic stresses than PSI, and PSII photoinhibition can protect PSI against photoinhibition by reducing the electron transport to PSI, however, PSI photoinhibition is more liable to occur just under chilling stress with low light due to the less inhibition on electron flow from PSII (Scheller and Haldrup, 2005). PSII photoinhibition reduced the electron flow to PSI in DH (**Figure 4C**), and consistently, the declined ΦPSII suggested that the actual photosynthetic electron transport from PSII was lowered after photosynthesis starting (**Figure 2E**). However, the inhibition on electron flow was not enough to protect PSI from photoinhibition (**Figure 4D**). Due to PSI photoinhibition, electrons could not be effectively driven to the acceptor side of PSI, and PSI oxidation would be shortened in the first 1 s red illumination. On the contrary, the declined electron flow from PSII could delay PSI re-reduction and prolong PSI oxidation. On the basis of the significant decrease in PSI oxidative amplitude (Inserted panels in **Figures 3B,D**), we can deduce that PSI photoinhibition was more severe than PSII photoinhibition in DH upon salt stress. As a result, the coordination between PSI and PSII was destroyed. In contrast, PSI and PSII photoinhibition in TH did not occur under salt stress in spite of the remarkable decrease in CO_2_ fixation (**Figure 4D**), because the increased NPQ helped to dissipate the excess excitation energy and potentially limit ROS production (**Figure 2F**). In consequence, the normal coordination between PSI and PSII was maintained in TH under salt stress. Similarly, normal PSI and PSII capacity was maintained in the halophyte *Thellungiella* under salt stress at 500 mM NaCl, but the effects on photosynthetic CO_2_ fixation and NPQ were not significant, indicating that its photosystem might not be endangered by the excess excitation energy (Stepien and Johnson, 2009). In agreement with the occurrence of photoinhibition, the extent of leaf lipid peroxidation in DH was significantly elevated by salt stress (**Table 1**), confirming the oxidative damage of ROS. Comparatively, no significant change was observed in leaf lipid peroxidation in TH after salt stress (**Table 1**), and this result was in accordance with the insignificant oxidative effects on photosystem.

Na^+^ is the primary toxic component for plants upon salt stress (Munns and Tester, 2008). Na^+^ can inhibit CO_2_ fixation by inducing negative effects on Rubisco, lead to the increase of ROS generation and irreversibly inactivate PSII and PSI (Allakhverdiev et al., 2000; Murata et al., 2007; Oukarroum et al., 2015). Thus, salt sensitive plant varieties tend to accumulate more Na^+^ in the leaf under salt stress and exhibit severe toxic symptoms (Chen et al., 2013; Aparicio et al., 2014). As an underlying reason for the higher salt tolerance, TH accumulated less Na^+^ in the leaf in contrast to DH under salt stress (**Table 1**). Consistently, salt-induced decrease in CO_2_ fixation was lower in the leaves of TH (**Figure 2D**), and as a result, PSII and PSI photoinhibition was not induced in line with no significant changes in lipid peroxidation (**Table 1**; **Figure 4**). It has been proposed that the divergence between salt tolerant and salt sensitive plants originates in the control of uptake and internal Na^+^ transport (Bojorquez-Quintal et al., 2014; Maathuis et al., 2014). Accordingly, we revealed that the lower leaf Na^+^ concentration in TH depended on the greater elevation of root Na^+^ extrusion and restriction of Na^+^ transport from root to leaf (**Table 1**).

## Conclusion

TH maintained normal PSII and PSI coordination by preventing photoinhibition and exhibited higher leaf photosynthetic activity than DH under salt stress. The higher leaf photosynthetic activity which contributed to biomass accumulation in TH might be ascribed to the lower ionic toxicity of Na^+^.

## Conflict of Interest Statement

The authors declare that the research was conducted in the absence of any commercial or financial relationships that could be construed as a potential conflict of interest.

## Abbreviations

CE: carboxylation efficiency
Ci: intercellular CO_2_ concentration
DH: diploid honeysuckle
ETo/ABS: quantum yield for electron transport
Fv/Fm: the maximal quantum yield of PSII
g_s_: stomatal conductance
MDA: malondialdehyde
MR/MR_0_: the maximal photochemical capacity of PSI
NMT: non-invasive micro-test technique
NPQ: non-photochemical quenching
OEC: oxygen-evolving complex
Pn: Photosynthetic rate
PSI: photosystem I
PSII: photosystem II
ΦPSII: actual photochemical efficiency of PSII
ROS: reactive oxygen species
Rubisco: ribulose-1,5-bisphosphate carboxylase/oxygenase
TH: tetraploid honeysuckle
W_k_: variable fluorescence intensity at K step.
